# The LBD Transcription Factor *ZmLBD33* Confers Drought Tolerance in Transgenic Arabidopsis

**DOI:** 10.3390/plants14091305

**Published:** 2025-04-25

**Authors:** Jing Xiong, Xin Mi, Lijuan Du, Xianqiu Wang

**Affiliations:** 1 Institute for Advanced Study, Chengdu University, Chengdu 610106, China; dulijuan@cdu.edu.cn; 2 School of Life Sciences, Sun Yat-sen University, Guangzhou 510275, China; mixin@mail2.sysu.edu.cn; 3 School of Modern Agriculture, Meishan Vocational and Technical College, Meishan 620010, China

**Keywords:** Lateral Boundary Domain (LBD), drought stress, hydrogen peroxide (H_2_O_2_)

## Abstract

Drought stress severely impacts maize productivity, necessitating the exploration of molecular mechanisms underlying drought responses. In maize, while Class I members of the LBD family have been extensively studied for their essential functions in developmental regulation and environmental stress responses, the potential involvement of Class II LBD genes in abiotic stress tolerance mechanisms remains poorly characterized. This study characterizes *ZmLBD33*, a maize Class II LBD gene, to elucidate its role in drought responses. Promoter analysis identified ABA-responsive cis-elements (AREB); *ZmLBD33* expression was strongly induced in roots under drought and ABA treatments, localized to the nucleus, and exhibited dimerization via yeast two-hybrid despite lacking intact leucine zipper motifs. *ZmLBD33*-overexpressed plants showed later germination, shorter roots, and decreased survival rates than wild-type plants under osmotic stress and soil drought. Compared to wild-type plants, *ZmLBD33*-overexpressed plants showed significantly faster water loss, a greater stomatal density, and reduced stomatal closure efficiency. Histochemical analysis using DAB and NBT showed attenuated reactive oxygen species accumulation in transgenic Arabidopsis overexpressing *ZmLBD33*. Quantitative enzymatic activity analyses further indicated that SOD and POD levels were significantly elevated in *ZmLBD33*-overexpressing plants compared to wild-type plants. These findings indicate that *ZmLBD33* negatively regulates drought tolerance by modulating stomatal aperture and H_2_O_2_ signaling. This study highlights the divergent roles of Class II LBD genes in stress adaptation and positions *ZmLBD33* as a potential target for engineering drought-resilient crops.

## 1. Introduction

Drought is one of the most important abiotic stresses that limit crop yields. It can inhibit the normal growth of plants, disrupt the water and ion balance within plants, cause stomata to close, reduce photosynthetic efficiency, increase the level of reactive oxygen species, damage the membranes of organelles, lead to metabolic disorders, decrease the accumulation of organic substances in plants, and even cause the premature death of plants [1,2,3]. Maize (*Zea mays*) has a wide range of applications in industrial and agricultural production and is an important raw material integrating food, industrial, and feed uses. The annual total output of maize in the world accounts for about 35% of the global total grain output [4]. However, due to the impact of global climate change and land desertification [5], the maize planting areas are affected by drought, resulting in a decrease in maize yield and a decline in quality. Therefore, understanding the response mechanisms of plants to drought stress, especially the molecular mechanisms of transcription factors under drought stress, and exploring the metabolic and signal transduction pathways of upstream and downstream gene regulation in depth are of great significance for enhancing maize’s adaptation to drought and water utilization efficiency, breeding drought-resistant varieties and ensuring food security and sustainable development of society.

*LBD* genes are a plant-specific class of transcription factors, with their proteins consisting of an N-terminal LOB domain and a variable C-terminal region [6]. The LOB domain contains a canonical CX2CX6CX3C motif, a conserved Gly-Ala-Ser (GAS) box, and a leucine zipper-like motif. The CX2CX6CX3C motif possesses DNA-binding activity, while the GAS box harbors a conserved proline residue [7,8]. The leucine zipper-like motif LX6LX3LX6L is implicated in protein dimerization and mediates interactions between LBD proteins and other proteins [9]. Based on the presence or absence of the leucine zipper-like motif in the LOB domain, *LBD* genes are categorized into two classes: Class I (containing the leucine zipper-like motif) and Class II (lacking this motif) [6]. Most members of the *LBD* gene family belong to Class I, which can form homodimers or heterodimers. However, Class II proteins lack the leucine zipper-like structure and cannot form coiled-coil configurations, distinguishing them structurally from Class I proteins [10].

The *LBD* gene family is unique to higher plants and plays crucial roles not only in plant growth and development but also in integrating developmental changes in response to plant hormone signals or environmental cues [8,10,11]. In Arabidopsis (*Arabidopsis thaliana*), *AtAS2 (At LBD6)* regulates the bilateral symmetry development of leaves into flat leaf blades [12,13]. AtAS2 can interact with AtAS1 to form a heterodimer that acts on the cis-regulatory elements of the *BP (KNAT1)* promoter, suppressing *KNAT1* expression activity and promoting leaf primordium development [14]. *AtLBD10* modulates ROS homeostasis by transcriptionally activating genes crucial for flavonol biosynthesis to maintain pollen tube growth and integrity [15]. In maize leaf primordia, LBD protein have been found to suppress the expression of *KNOX* genes [16]. The maize IG1 (indeterminate gametophyte1) inhibits female gametophyte development [17] and interacts with RS2 (the ortholog of AtAS1) to influence tassel branching [18]. In rice (*Oryza sativa* L.) and maize, *LBD* genes such as *CRL1* (*ARL1*) and *RTCS* are expressed in root cap primordia, regulating root cap development under auxin regulation [19,20,21]. Additionally, the auxin signaling component IAA13 cooperates with ARF19 to promote *LBD1-8* transcription, controlling constitutive aerenchyma formation in seed coat cells and pericycle-mediated lateral root development [22]. Goh et al. (2019) [23] demonstrated that auxin-mediated regulation of *PUCHI*, a key regulator of lateral root primordia and a downstream target of *AtLBD16*, depends on the IAA14-ARF7-ARF19 signaling pathway. PagLBD3 enhances secondary growth in poplar by modulating cambium cell differentiation into phloem through regulation of key xylem development genes, including *PXY* (*Phloem Intercalated with Xylem*), *WOX4* (*Wuschel Related Homeobox4*), *SND1-B2* (*Secondary Wall-associated NAC Domain 1s*), and *VND6-B1* (*Vascular-related NAC domain 6s*) [24]. Beyond growth regulation, *LBD* genes participate in plant responses to biotic and abiotic stresses [11]. Kong et al. (2020) [25] revealed that the pathogen Pto DC3000 significantly promotes lateral root development via the canonical auxin signaling pathway IAA14-ARF7/19-LBD16/18, highlighting *LBD* genes in modulating pathogen invasion through lateral roots and providing insights into root–microbe interactions. ABA, a critical hormone in drought response, regulates lateral root branching and root architecture via AtLBD14-mediated ABA signaling [26,27]. AtLBD15 directly binds to the promoter of the ABA signaling gene *AtABI4* to activate its expression, leading to stomatal closure, reduced water loss, and enhanced drought tolerance in overexpressing plants [28]. In rice, OsLBD12-1 binds to the *AGO10* promoter to repress its expression, resulting in developmental retardation, twisted leaves, abnormal anthers, and smaller shoot apical meristems (SAM). Under salt stress, OsLBD12-1 exerts stronger suppression of *AGO10* [29]. In maize, *ZmLBD5* modulates drought tolerance by regulating ABA and GA levels [30].

In contrast, reports about Class II members remain limited. This study explores the function of *ZmLBD33*, a Class II transcription factor, in mediating drought stress responses. Overexpression of *ZmLBD33* in Arabidopsis resulted in a drought-sensitive phenotype characterized by significantly delayed seed germination, impaired root development, and compromised seedling survival rates under osmotic stress and soil drought conditions. *ZmLBD33* functions as a negative regulator of drought tolerance involving stomatal aperture and H_2_O_2_-mediated signaling pathways, which identified *ZmLBD33* as a promising genetic target for engineering drought-resistant crops through modulation of water conservation strategies.

## 2. Results

### 2.1. Analysis of the Gene Structure and Expression of ZmLBD33

**Figure 1:**
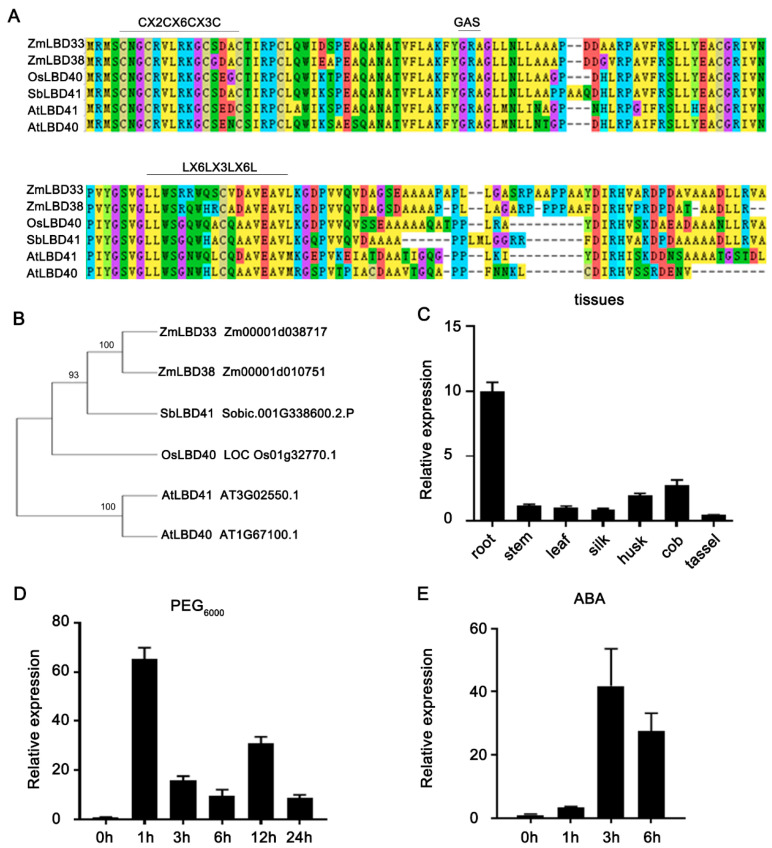
Sequence analysis and expression pattern of *ZmLBD33.* (**A**) Alignment of conserved LBD domain sequences between ZmLBD33 and its homologous genes in other species. (**B**) Phylogenetic relationships of ZmLBD33 proteins from maize and selected species. The phylogenetic tree was constructed by utilizing the Neighbor-Joining (NJ) approach with 1000 bootstrap replications. Expression pattern of *ZmLBD33* in (**C**) different tissues, (**D**) drought stress, and (**E**) abscisic acid (ABA) treatment. Expression level changes in RNA transcripts were calculated by the 2^−∆∆Ct^ method with *Zme1F1α* and *Zm18S* as internal control. All bars represent means ± SD (*n* = 3).

In this study, the Class II *ZmLBD33* gene was isolated from the maize inbred line B73, exhibiting a full-length coding region of 963 base pairs that translates into a 320-residue polypeptide. Bioinformatic analyses demonstrated the predicted molecular weight of 34.03 kDa and theoretical isoelectric point (pI) of 6.05 for this protein. Multiple sequence alignment identified the characteristic CX_2_CX_6_CX_3_C zinc finger motif essential for DNA binding, while lacking conserved GAS domains and containing truncated LX_6_LX_3_LX_6_L helices—characteristic features differentiating Class I and II LBD members (Figure 1A). Based on the amino acid sequence of ZmLBD33, homologous sequences in other species, including Arabidopsis (AtLBD40 and AtLBD41), *Oryza sativa* (OsLBD40), and *Sorghum bicolor* (SbLBD41), were screened by using the Phytozome Database V13 of the BLAST program (Figure 1A). The phylogenetic analysis showed that ZmLBD33 was very similar to SbLBD41 from Sorghum bicolor (Figure 1B).

Motivated by the critical regulatory function of promoters in transcriptional control, we performed cis-element analysis on the 1 kb upstream regulatory region flanking the translation initiation codon of ZmLBD33. The *ZmLBD33* promoter contained some stress-regulatory motifs, quantitatively dominated by four hits ABRE (ABA-responsive element), three hits ARE (anaerobic induction element), three hits G-Box (light responsiveness element), and other light response elements (Table 1). ABRE plays a key role in ABA signaling pathway and participates in the response to drought stresses.

To validate *ZmLBD33*′s drought responsiveness, its transcript abundance was quantified in root and leaf tissues under drought stress or abscisic acid (ABA). The results showed that under drought and ABA treatment, the expression of *ZmLBD33* significantly increased in roots, especially after 12 h of drought treatment, reaching more than 50 fold (Figure 1D,E). These findings suggest *ZmLBD33* contributes functionally to maize drought resistance. To systematically characterize its developmental roles, spatiotemporal expression patterns were profiled across 11 tissues representing key growth stages. qRT–PCR analysis demonstrated that *ZmLBD33* transcripts accumulated preferentially in root tissues, though detectable levels were observed in all examined maize organs (Figure 1C).

### 2.2. ZmLBD33 Is a Nucleus-Localized Protein and Could Form Dimers

**Figure 2:**
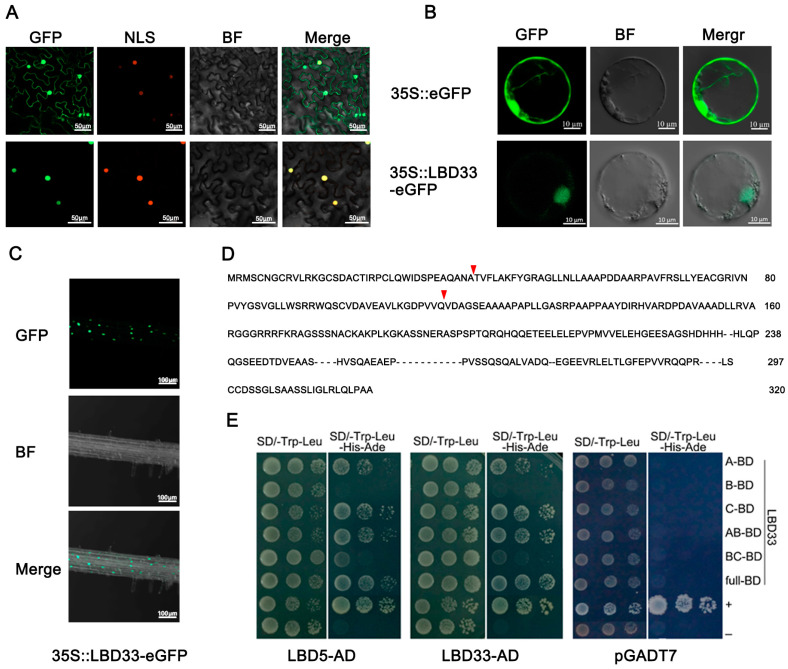
Subcellular localization and the ability to form dimers of ZmLBD33. (**A**,**B**) Subcellular localization of ZmLBD33 in tobacco leaves and maize protoplast. Bar = 50, 10 µm, respectively. (**C**) ZmLBD33 protein is localized in nuclei in Arabidopsis, Bar = 100 µm. (**D**) ZmLBD33 was truncated into three segments (**A**–**C**), with truncation sites marked by red triangles. (**E**) ZmLBD33 dimerization was tested in the Y2H Gold yeast strain. Transformed Y2H Gold strains were serially diluted and plated on non-selective (-T-L) or selective (-T-L-H-A) SD media. Imaging was performed after 3-day incubation. Fragments A (CX2CX6CX3C), B (GAS), and C (LX6LX3LX6L) correspond to distinct zinc finger motifs and C-terminal regulatory domains.

Subcellular localization analysis serves as a critical prerequisite for functional annotation of gene products. Subcellular compartmentalization of ZmLBD33 was analyzed using a 35S promoter-driven GFP fusion system, implemented through Agrobacterium-mediated transient expression in tobacco leaves and PEG-medicated transfection in maize protoplasts. The strong green fluorescence signal of 35S::GFP was mainly distributed in the nucleus and the cytoplasm, whereas the green fluorescence signal of 35S::ZmLBD33-GFP was observed in the nucleus, which completely overlapped with the red fluorescence signal of the nuclear localization signal (Figure 2A,B). To confirm the nuclear localization of ZmLBD33, transgenic plants overexpressing ZmLBD33 with a C-terminal GFP fusion were analyzed. Fluorescence microscopy revealed GFP signals specifically localized in root cell nuclei (Figure 2C). This result was further confirmed by the subcellular localization assay with the transient expression of ZmLBD33-GFP in tobacco leaves and in maize protoplasts, indicating that transcription factors play a regulatory role in the nucleus.

With GAS and leucine zipper domains being essential for the formation of dimers, their absence or incompleteness was tested in Class II member ZmLBD33 using the Yeast two hybrid method. The intact ZmLBD33 protein and five truncated variants (A, B, C, AB, BC) were screened for homotypic (self-interaction) and heterotypic (ZmLBD5, another LBD member) binding capacities. Fragments A, B, and C represent the N-terminal C-block (CX2CX6CX3C), the GAS and LX6LX3LX6L coiled-coil motifs, and the C-terminal domain, respectively (Figure 2D). Although ZmLBD33 has incomplete GAS and leucine zipper domains, it can still form homo—or heterodimers.

### 2.3. ZmLBD33 Overexpression Compromised Drought Tolerance in Arabidopsis

**Figure 3:**
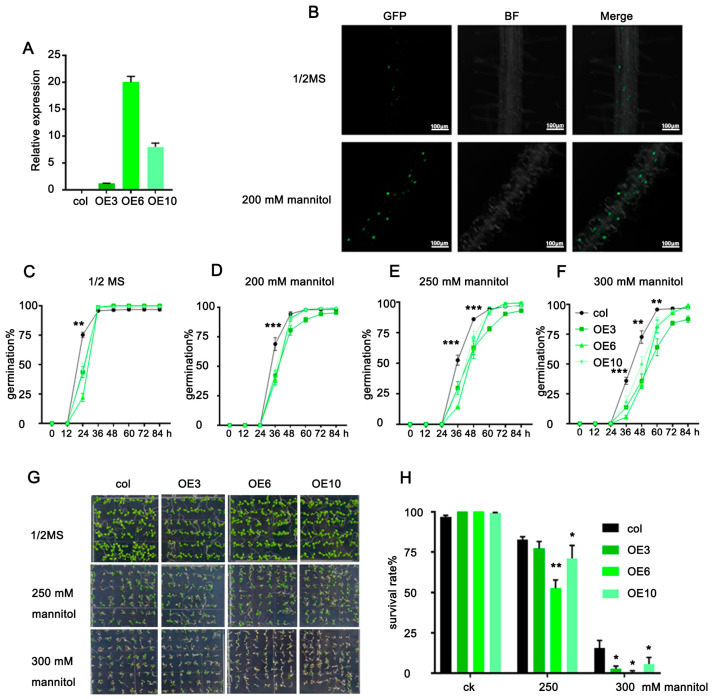
Germination and cotyledon greening rate of *ZmLBD33* transgenic Arabidopsis under mannitol stress. (**A**) Expression level in *ZmLBD33*-transgenic Arabidopsis lines. (**B**) Protein expression level of ZmLBD33 under 200 mM mannitol stress based on the GFP fluorescence signal. (**C**–**F**) Germination and (**G**,**H**) cotyledon greening rate of *ZmLBD33* transgenic Arabidopsis under 200 mM, 250 mM, 300 mM mannitol stress. Statistical significance was evaluated using one-way analysis of ANOVA. *, *p* < 0.05, **, *p* < 0.01, ***, *p* < 0.001. Error bars signify standard deviations, (*n* ≥ 3).

To investigate *ZmLBD33*′s biological role, we generated 11 Arabidopsis transgenic lines overexpressing this gene via Agrobacterium-mediated floral dip transformation. Three T3 homozygous lines (OE3, OE6, OE10) exhibiting strong overexpression (Figure 3A) were prioritized for phenotypic analyses. Confocal microscopy revealed osmotic stress-induced accumulation of ZmLBD33-GFP fusion proteins under 200 mM mannitol treatment (Figure 3B), suggesting drought-responsive regulation of its stability or turnover.

The overexpressed lines and wild-type seeds were sown on 1/2 Murashige and Skoog medium with four mannitol concentrations (0, 200, 250, and 300 mM), and the germination rate was counted every 12 h. The germination of *ZmLBD33* overexpressed lines and wild type was normal under normal conditions. However, elevated osmotic stress induced by incremental mannitol concentrations revealed progressively delayed germination kinetics in *ZmLBD33* transgenic lines compared to wild-type controls, as quantitatively demonstrated in the temporal germination profiles (Figure 3C–F). After 3 days of germination, albino seedlings became obvious at 250 mM and 300 mM mannitol. Transgenic lines overexpressing seedlings exhibited a significantly reduced rate of cotyledon greening compared to wild-type seedlings (Figure 3G,H). This indicated that *ZmLBD33* overexpression was sensitive to osmotic stress in Arabidopsis.

**Figure 4:**
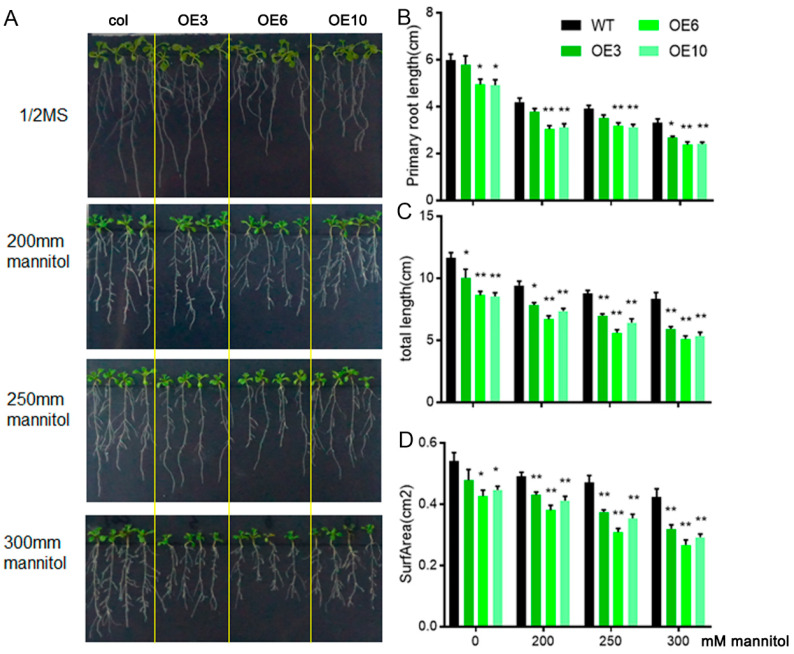
Development and osmotic tolerance in *ZmLBD33* transgenic Arabidopsis. (**A**) Comparative growth phenotypes of WT and *ZmLBD33* transgenics cultured on 1/2MS medium under differential mannitol concentrations (0, 200, 250, and 300 mM). (**B**–**D**) Root architectural quantification of root systems in *ZmLBD33* transgenics and wild-type seedlings under osmotic stress, assessing primary root length, total root length, and root surf area. Error bars indicate standard deviation (SD) derived from ≥12 biologically independent replicates. * *p* < 0.05, ** *p* < 0.01.

To further characterize the morphological responses of wild-type and transgenic seedlings to mannitol-induced osmotic stress, multigenic plantlets were first cultivated on 1/2 MS substrate for 5 days, followed by secondary exposure to vertically oriented agar plates containing incremental mannitol concentrations (0, 200, 250, 300 mM). Phenotypic evaluations were systematically performed after a 7-day stress acclimatization period. With increasing mannitol concentrations, the rosette leaf size, primary root length, total root length, and root surface area of both *ZmLBD33*-transgenic and wild-type seedlings exhibited a reduction. However, these morphological parameters were significantly more pronounced in *ZmLBD33*-transgenic seedlings compared with the wild type (Figure 4). These findings suggest that the *ZmLBD33* transgenic seedlings are more sensitive to osmotic stress induced by mannitol, as evidenced by their impaired growth and development under such conditions.

**Figure 5:**
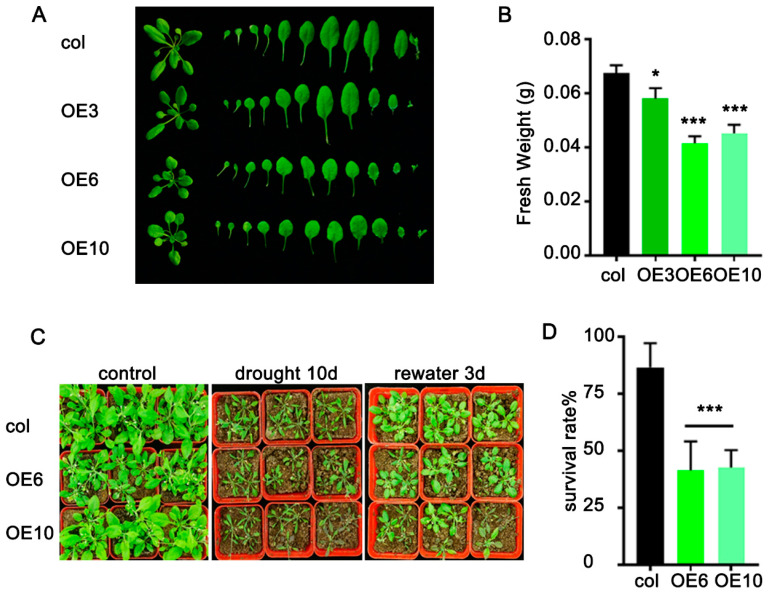
Phenotype and survival of *ZmLBD33* transgenic seedlings in soil under drought stress. (**A**,**B**) Phenotype and fresh weight of *ZmLBD33*-overexpressed seedlings in normal condition (**C**,**D**) Survival rate under drought stress. Statistical significance was evaluated using one-way analysis of ANOVA. *, *p* < 0.05, ***, *p* < 0.001. Error bars signify standard deviations, (*n* ≥ 12).

To further investigate the role of *ZmLBD33* in drought resistance in Arabidopsis, transgenic and wild-type seedlings grown on normal medium for 7 days were transplanted into soil and cultivated for one month. Subsequently, water was withheld for 10 days to induce drought stress. Following rewatering, the majority of *ZmLBD33*-transgenic seedlings exhibited wilting, while wild-type seedlings remained turgid with their leaves retaining green coloration (Figure 5C). Under normal growth conditions, the overexpression of *ZmLBD33* in Arabidopsis resulted in inhibited seedling growth, as evidenced by a significant reduction in fresh weight compared with the wild-type seedlings (Figure 5A,B). Following drought treatment and subsequent rewatering, the survival rate of Arabidopsis seedlings overexpressing *ZmLBD33* was notably lower compared to that of wild-type seedlings (Figure 5D). These findings demonstrate that the overexpression of *ZmLBD33* in Arabidopsis adversely affects plant growth and development and increases the susceptibility of Arabidopsis plants to drought stress.

### 2.4. ZmLBD33 Promoted Water Loss Rate Through the Stomatal Density and Aperture

**Figure 6:**
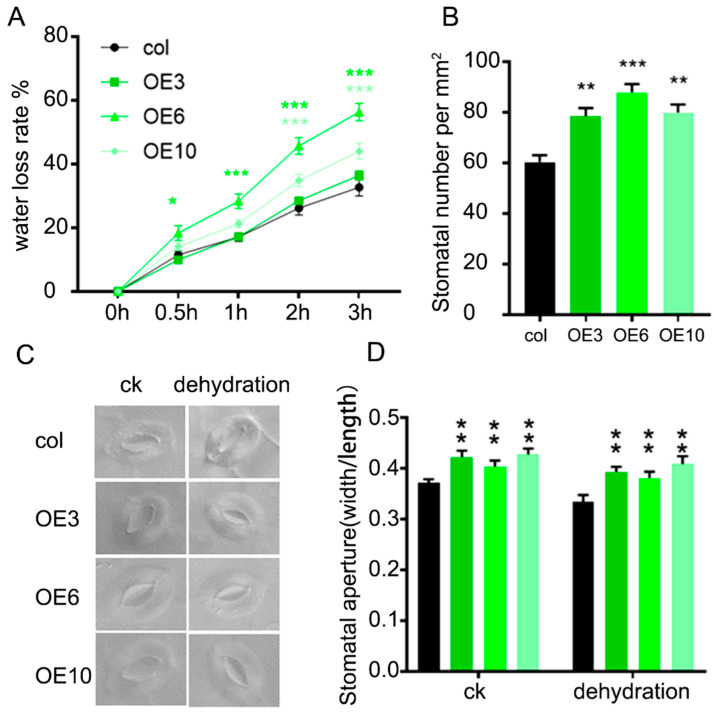
Water loss and stomatal features in *ZmLBD33*-transgenic Arabidopsis leaves. (**A**) Leaf water loss rate. (**B**) Stomatal density on the fourth leaf. (**C**,**D**) Stomatal aperture on the fourth leaf after detachment 1 h. Statistical significance was evaluated using one-way analysis of ANOVA. *, *p* < 0.05, **, *p* < 0.01, ***, *p* < 0.001. Error bars signify standard deviations, (*n* ≥ 12).

To explore the underlying mechanism behind the drought sensitivity exhibited by *ZmLBD33*-overexpressed seedlings, we measured the water loss rate of detached leaves. Following 60 min of hydropenic stress, the results revealed a significant increase in transpirational water loss from excised foliage of *ZmLBD33*-overexpression lines relative to wild-type (Figure 6A). This finding implied that *ZmLBD33*-overexpressed seedlings depleted soil moisture more rapidly than wild-type seedlings, consequently leading to earlier wilting. As stomata are the main channels through which water evaporation takes place, we conducted an analysis of the number and apertures of stomata on the abaxial surface of the leaves. The results indicated that both the stomatal number and stomatal aperture of *ZmLBD33*-overexpressed seedlings were greater than those of wild-type seedlings (Figure 6B–D). Thus, the overexpression of *ZmLBD33* in Arabidopsis led to an increase in both stomatal number and aperture under drought conditions, thereby enhancing the plant’s sensitivity to drought.

### 2.5. Overexpression of ZmLBD33 Augmented the Activity of Antioxidant Enzymes and Prevented the Accumulation of ROS in Arabidopsis

**Figure 7:**
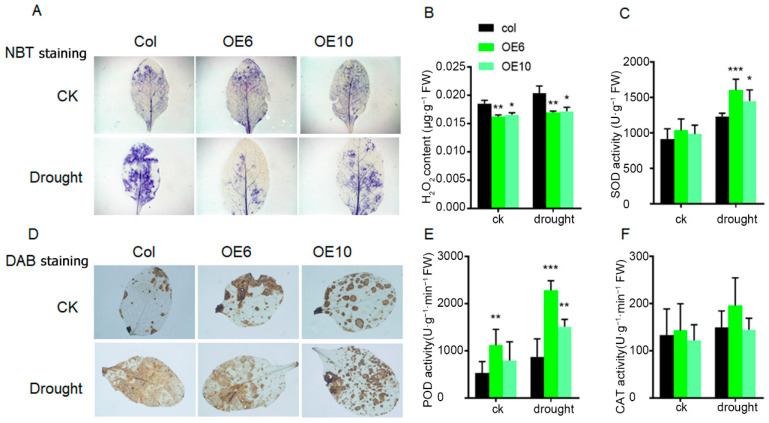
Analysis of reactive oxygen species staining and physiological indexes in *ZmLBD33*-Transgenic Arabidopsis. (**A**,**D**) NBT and DAB staining of leaves for H_2_O_2_ in *ZmLBD33*-overexpressed Arabidopsis under drought stress treatments. (**B**) H_2_O_2_ content measurement in *ZmLBD33*-overexpressed Arabidopsis with KI method; SOD activity (**C**), POD activity (**E**), and CAT activity (**F**) in leaves in ZmLBD33-overexpressed Arabidopsis. Statistical significance was evaluated using one-way analysis of ANOVA. *, *p* < 0.05, **, *p* < 0.01, ***, *p* < 0.001. Error bars signify standard deviations, (*n* = 3).

Hydrogen peroxide (H_2_O_2_), a key signaling molecule in plant stress responses, plays a pivotal role in mediating drought stress adaptation through its regulation of stomatal closure to minimize water loss. To investigate whether the observed sustained stomatal opening in *ZmLBD33*-overexpressing seedlings correlated with altered H_2_O_2_ accumulation, we performed histochemical staining and quantitative analysis using one-month-old transgenic and wild-type plants subjected to a 10-day drought treatment. Histochemical analysis utilizing nitroblue tetrazolium (NBT) and 3,3′-diaminobenzidine (DAB) staining revealed significantly lighter staining intensity in transgenic leaves compared with wild-type under drought stress conditions (Figure 7A,D). Under both normal and drought conditions, quantitative measurement of H_2_O_2_ content in aerial tissues using the potassium iodide method confirmed that *ZmLBD33*-overexpressing plants maintained lower H_2_O_2_ levels than wild-type plants (Figure 7B).

Given the well-established relationship between reactive oxygen species (ROS) homeostasis and stress tolerance, mediated through antioxidant enzyme activities, we further analyzed the enzymatic activities of key antioxidant enzymes. *ZmLBD33*-overexpressing seedlings exhibited significantly enhanced superoxide dismutase (SOD) and peroxidase (POD) activities compared to wild-type (Figure 7C,E). However, catalase (CAT) activity remained unchanged between transgenic and wild-type plants (Figure 7F), indicating a specific regulatory pattern of antioxidant enzyme activities in response to *ZmLBD33* overexpression.

### 2.6. Drought-Inducible Genes Were Transcriptionally Suppressed in ZmLBD33-Overexpressing Arabidopsis

**Figure 8:**
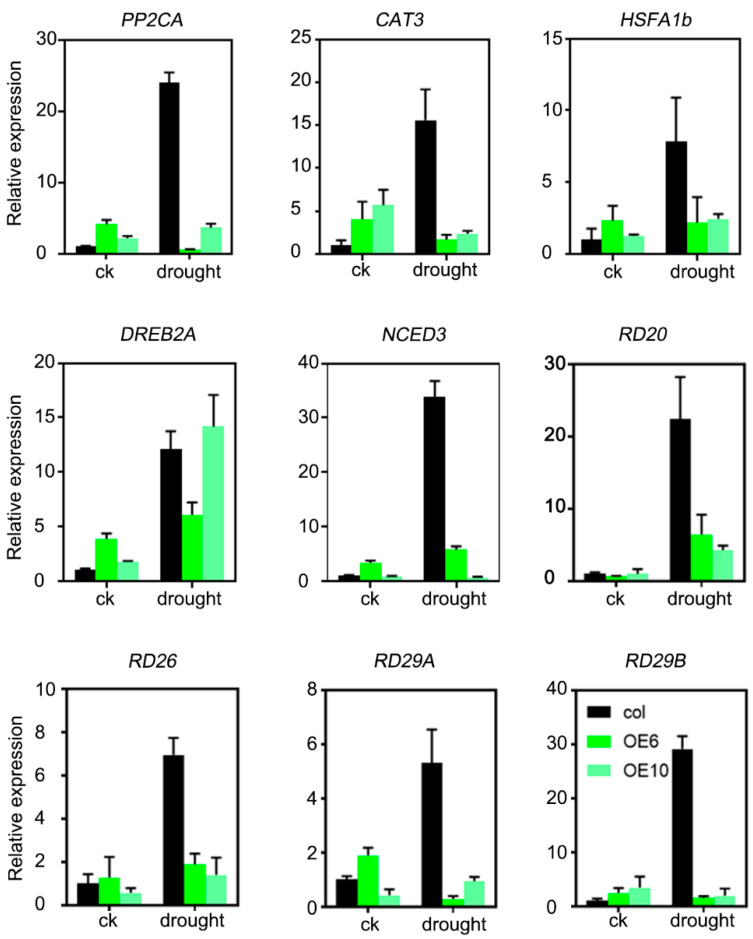
Relative expression level of drought stress-related genes in *ZmLBD33* transgenic Arabidopsis and wild type. CK represented plants growing under normal conditions; drought indicated plants growing under drought stress until leaf wilting. Black column represents wild type seedlings and green column represents transgenic seedlings.

The expression levels of drought-responsive genes serve as a reliable indicator for evaluating plants’ ability to respond to drought stress. In this study, we selected nine well-characterized genes (*AtPP2CA, AtCAT3, AtHSFA1b, AtDREB2A, AtNCED3, AtRD20, AtRD26, AtRD29A, and AtRD29B*) that are known to be significantly upregulated under drought conditions. Our experimental analysis compared the relative expression levels of these genes between transgenic seedlings and wild-type plants under both normal and drought stress conditions. No significant differences in gene expression levels were observed under normal growth conditions. However, when subjected to drought stress, both transgenic and wild-type plants exhibited substantial upregulation of all nine drought-responsive genes. Interestingly, the *ZmLBD33*-transgenic plants showed consistently lower expression levels of these genes compared to their wild-type counterparts (Figure 8). These findings suggest that *ZmLBD33* plays a regulatory role in modulating the expression of drought-related genes under stress conditions, acting as a negative regulator of the plant’s drought stress response.

## 3. Discussion

The *LBD* gene family, a group of plant-specific transcription factors, is classified into Class I and Class II members based on the presence or absence of the GAS domain and leucine zipper motif. With the increasing availability of genomic data across various plant species, the distribution, gene structure, expression patterns, and functional roles of *LBD* genes in plant growth and development have been extensively studied [10,31,32,33]. Class I *LBD* genes are predominantly involved in diverse developmental processes, including root growth, leaf expansion, pollen development, plant regeneration, photomorphogenesis, pathogen responses, and secondary cell wall formation [8,11,34]. In contrast, the functional characterization of Class II *LBD* genes remains relatively limited. Current studies suggest that Class II members play roles in anthocyanin biosynthesis, nitrogen metabolism, and gibberellin (GA) responses in Arabidopsis and maize [30,34,35], flowering time regulation, and nitrogen metabolism in rice [36], and root development and auxin signaling in Medicago [37]. These findings highlight the potential role of Class II *LBD* genes in integrating hormonal signaling and environmental responses.

Promoter analysis of *LBD* genes in wild tea plants (*Camellia sinensis*) revealed the presence of cis-acting elements responsive to cold stress, plant defense, drought, and jasmonic acid signaling, suggesting their involvement in abiotic and biotic stress responses [33]. Similarly, in ramie (*Boehmeria nivea*), *LBD* genes were shown to confer resistance to abiotic stresses, particularly drought and high temperature, with distinct expression patterns under these conditions [3]. In this study, we identified that the Class II *LBD* genes *ZmLBD33* are ubiquitously expressed across various tissues (Figure 1C), indicating their potential involvement in multiple aspects of plant growth and development. Under drought conditions, the expression of *ZmLBD33* was significantly upregulated in roots (Figure 1D). Furthermore, their expression was strongly induced by abscisic acid (ABA) (Figure 1E), a key hormone in drought stress responses, suggesting their participation in drought stress signaling pathways.

Previous studies have established that Class I *LBD* transcription factors play pivotal roles in plant organogenesis, including embryogenesis, root architecture establishment, leaf morphogenesis, and inflorescence patterning [12,13,17,38]. In *Arabidopsis thaliana*, spatial expression patterns of *AtASL4* (*AtLBD4*) at the leaf primordia-shoot apical meristem boundary demonstrated its regulatory function in foliar development through boundary formation control [38]. The AS2/AtLBD6 protein acts as a transcriptional repressor restricting cell proliferation in leaf adaxial domains, with loss-of-function mutants exhibiting defective leaf expansion due to impaired dorsoventral patterning [13,17]. Functional analyses reveal that AtLBD16 and AtLBD18 coordinate auxin signaling pathways to regulate callus formation and root regeneration processes [9,39]. In maize, the LBD member indeterminate gametophyte1 (IG1) exhibits dual functionality: it suppresses *knotted1-like homeobox* (*KNOX*) gene expression to establish ligule/auricle boundaries during leaf development while simultaneously regulating reproductive development by limiting male inflorescence branching and ensuring female gametophyte viability [17]. Beyond developmental regulation, emerging evidence highlights Class I LBDs’ involvement in abiotic stress responses. *AtLBD15* demonstrated functional pleiotropy by enhancing drought tolerance through dual mechanisms: modulating abscisic acid (ABA) signaling pathways to induce stomatal closure and activating reactive oxygen species (ROS) scavenging systems [28]. Transgenic Arabidopsis overexpressing *AtLBD15* exhibited upregulated stress-responsive markers (*RD29A*, *COR15A*) and superior drought resilience [28]. Similarly in rice, OsLBD12-1 confers salinity tolerance through transcriptional regulation of *AGO10*, a core component of the RNA-induced silencing complex (RISC). Under NaCl stress, OsLBD12-1 binds to the *AGO10* promoter to downregulate its expression, thereby enhancing cellular ion homeostasis [29]. Intriguingly, functional conservation extends across LBD classes. While Class II members like *ZmLBD5* in maize primarily regulate drought responses via ABA biosynthesis upregulation (e.g., activating *ZmNCED3* expression in transgenic lines), certain Class II proteins exhibit developmental roles overlapping with Class I functions [30,40]. In Arabidopsis and rice, orthologous clade members *LBD37/38/39* exhibit functional synergy in negatively regulating nitrogen assimilation pathways through transcriptional suppression mechanisms [34,41]. This regulatory paradigm extends to legume systems, where heterologous expression of *MsLBD48* was shown to transcriptionally repress core nitrate assimilation components, specifically targeting nitrate transporter genes (*NRT2.1/2.2*) and nitrite reductase isoforms (*NIA1/2*) in Arabidopsis [42]. Functional characterization in Malus (*Malus pumila Mill*) domestica revealed that *MdLBD13* transcriptionally represses both anthocyanin biosynthesis and nitrogen assimilation efficiency through modulation of flavonoid metabolic networks [43]. Whereas *ZmLBD33*, despite its classification as a Class II gene, modulates both organogenesis and drought responses in transgenic Arabidopsis, mirroring the functionality of Class I proteins (Figure 4 and Figure 5). This functional convergence between phylogenetically distinct *LBD* classes underscores the necessity for comprehensive characterization of Class-II members in plant development and stress adaptation mechanisms.

To functionally characterize *ZmLBD33*, we utilized *Arabidopsis thaliana* as a heterologous expression system, capitalizing on its well-established advantages including rapid life cycle, genetic tractability, and high transformation efficiency. This model plant has been extensively validated for functional studies of stress-responsive genes across cereal crops such as maize, sorghum, and wheat, particularly in drought tolerance research [44,45,46]. Previous successful examples include the enhanced drought and heat tolerance in Arabidopsis through heterologous expression of rice *OsDREB2B* and maize *ZmDREB* transcription factors, which functioned by activating conserved stress-responsive pathways [45,46]. Similarly, the drought-tolerant phenotype observed in *ZmPP2C-A10*-overexpressing Arabidopsis was subsequently confirmed in maize, demonstrating the predictive value of this cross-species approach [47]. In our investigation, heterologous expression of *ZmLBD33* in Arabidopsis elicited distinct drought-sensitive phenotypes. Transgenic lines displayed significantly reduced cotyledon greening rates under mannitol-induced osmotic stress and diminished survival rates under progressive soil drought compared to wild-type controls (Figure 3). Complementary physiological analyses revealed three key water-related deficiencies: accelerated water loss rates in detached leaves, increased stomatal density on abaxial leaf surfaces, and impaired stomatal closure regulation under dehydration stress (Figure 6). These convergent findings suggest that *ZmLBD33* acts as a negative regulator of drought tolerance through dual mechanisms: promoting excessive transpirational water loss via stomatal developmental and operational alterations, while simultaneously compromising cellular water retention capacity.

H_2_O_2_, a key signaling molecule in stomatal closure, was found to accumulate at lower levels in *ZmLBD33*-overexpressing plants compared with wild-type plants under normal and drought conditions (Figure 7B). Although the activities of SOD and POD were significantly elevated in transgenic plants, catalase (CAT) activity remained unchanged, indicating that the regulation of drought stress by *ZmLBD33* is not primarily mediated through antioxidant pathways but rather through H_2_O_2_ signaling and stomatal regulation. Furthermore, the expression levels of well-characterized drought-responsive genes, such as *AtPP2CA, AtCAT3, AtHSFA1b, AtDREB2A, AtNCED3, AtRD20, AtRD26, AtRD29A*, and *AtRD29B*, were significantly reduced in transgenic plants relative to wild-type plants under drought stress (Figure 8). This suggests that *ZmLBD33* may directly or indirectly modulate the expression of these genes, thereby influencing drought stress responses.

## 4. Method and Materials

### 4.1. Plant Materials and Growth Conditions

The maize inbred line B73 was employed to investigate the expression pattern of *ZmLBD33* across different tissues and under various stress treatments. Root, stem, leaf, silk, husk, cob, and tassel tissues were harvested at the V13 developmental stage, immediately frozen in liquid nitrogen, and stored at −80 °C. Each biological replicate consisted of three seedlings with uniform growth. Two-leaf-stage seedlings were transplanted into Hoagland’s nutrient solution in a greenhouse maintained at 28 °C under a 14 h light/10 h dark photoperiod. After growing to the three-leaf stage, plants were subjected to stress treatments: 20% (*w*/*v*) polyethylene glycol 6000 (PEG6000) or 10 µM abscisic acid (ABA). For PEG6000 treatment, root and leaf samples were collected at 0 h, 1 h, 3 h, 6 h, 12 h, and 24 h post-treatment. For ABA treatment, root samples were collected at 0 h, 1 h, 3 h, and 6 h. All samples were flash-frozen in liquid nitrogen and stored at −80 °C. Each experiment included three biological replicates. Primers specific to the target genes for expression pattern analysis are listed in Table S1.

### 4.2. Sequence Analysis and Phylogenetic Tree Construction

The sequence of *ZmLBD33* was obtained from MaizeGDB (https://www.maizegdb.org/, accessed on 5 May 2017). The homologous protein sequence of ZmLBD33 was retrieved from the Phytozome database (https://phytozome-next.jgi.doe.gov/, accessed on 1 July 2024) and protein alignment analysis was carried out using ClustalW 2.1. Plant CARE (http://bioinformatics.psb.ugent.be/webtools/plantcare/html/, accessed on 7 July 2024) was used to analyze the abiotic-stress-related cis-elements of the promoter sequence. The phylogenetic tree was constructed with the Neighbor-Joining method, and the test of the branches was calculated based on 1000 replications of bootstraps. Analyses were performed using MEGAX V11.

### 4.3. Subcellular Localization

The coding sequence (CDS) of *ZmLBD33* without the termination codon was inserted into the binary vector pCAMBIA2300—eGFP to generate the pCAMBIA2300—ZmLBD33—eGFP vector, which was driven by the CaMV35S promoter. The constructed vector was introduced into Arabidopsis and tobacco leaves via the agrobacterium-mediated method. Additionally, the plasmid was transformed into maize protoplasts using the PEG-mediated method. GFP fluorescence was examined using a laser confocal microscope (LSM800, Zeiss, Germany). The primers used in this experiment are listed in Supplementary Table S1.

### 4.4. RNA Extraction and Quantitative RT-qPCR Analysis

Total RNA was extracted from Arabidopsis or maize seedlings following the manufacturer’s protocol of the Plant Total RNA Isolation Kit (FOREGENE, Chengdu, China; Re—05014). To eliminate genomic DNA contamination, the RNA was treated with DNase I (Trans, Beijing, China; GD201—01) at 37 °C for 30 min. The PrimeScript RT reagent kit with a gDNA eraser (Takara, Kyoto, Japan; RR047A) was employed to synthesize cDNA, which served as templates for real-time PCR. Subsequently, qRT–PCR experiments were conducted using a Bio-Rad CFX96 real-time system (Bio-Rad, Hercules, CA, USA) with the SYBR Green Fast qPCR Mix Kit (ABclonal, Wuhan, China; RM21203) according to the manufacturers’ protocols. *AtACTIN8* and *AtUBQ10* were used as internal reference genes for Arabidopsis, while *ZmeF1α* and *Zm18S* were used for maize. The gene expression levels were calculated using the 2^−∆∆Ct^ method. Three independent biological replicates of each cDNA sample were performed to ensure accurate statistical analysis. All the primers used in the experiments are listed in Supplementary Table S1.

### 4.5. Transcriptional Activation Analysis and Y2H

The full-length and truncated forms of *ZmLBD33* were amplified and inserted into the pGBKT7 vector, while the full-length *ZmLBD5* and *ZmLBD33* were cloned into the pGADT7 vector. *ZmLBD33* (full-length/truncated) constructs were generated in pGBKT7, whereas full-length *ZmLBD5* and *ZmLBD33* were co-expressed via pGADT7. Then, the full-length and truncated forms of *ZmLBD33* were respectively transformed into Y2H strain together with ZmLBD5-pGADT7 and ZmLBD33-pGADT7, and cultured at 30 °C on SD-Trp/Leu and SD-Trp/Leu/His/Ade for 3–5 days. All of the specific primers were listed in Supplementary Table S1.

### 4.6. Generation of Transgenic Plants and Phenotypic Analysis

The coding sequence of *ZmLBD33* was cloned into the pCAMBIA2300-eGFP vector to generate the 35S::ZmLBD33-eGFP vector. The constructed vector was transformed into the wild-type Col-0 Arabidopsis using the agrobacterium-mediated floral dip method to obtain *ZmLBD33* overexpression plants. Homozygous plants were screened under 10 µg/mL neomycin (G418) conditions, and three high-expression lines were selected for further study. All the primers used in the experiments are listed in Supplementary Table S1.

Seeds were sterilized with 75% alcohol for 5 min, rinsed once with sterilized water, and then disinfected with 3% sodium hypochlorite containing 0.1% TritonX10 for 10 min. After washing 5–6 times with sterilized water, the seeds were sown on 1/2MS, 1/2MS + 200 mM mannitol, 1/2MS + 250 mM mannitol, and 1/2MS + 300 mM mannitol culture media for growth.

Germination rates were recorded every 12 h. A seed was considered germinated when the radicle protruded from the seed coat. After 5 days of germination, the greening rate of the seedlings was recorded under different stress conditions. Albino seedlings and non-germinated seeds were counted as dead plants, while green plants were considered normal, and the survival rate of the plants was calculated. Each sample had three biological replicates. Statistical analysis was performed using one-way ANOVA, and the mean values of each transgenic line were compared with those of the wild type.

Five-day-old seedlings with similar growth were vertically grown on 1/2 MS medium containing 0 mM, 200 mM, 250 mM, and 300 mM mannitol for one week. The primary root length was measured using ImageJ 1.45 software. Root lengths and surface areas were collected using an Epson 11,000XL root scanner (Epson, Nagano, Japan) and WinRHIZO pro2013. All experiments were repeated three times, and each biological replicate contained at least 12 seedlings.

Seven-day-old plants were transplanted into soil under short-day conditions (10 h light, 14 h dark, 25 °C) and grown for one month. Subsequently, watering was withheld for approximately 10 days, and photographs were taken. After re-watering for 3 days, photographs were taken again, and the survival rates were determined.

### 4.7. Water Loss Measurement

To measure the water loss rate, 12 one-month-old seedlings of each genotype were detached and placed on the laboratory bench. They were weighed at predefined time points (0 h, 0.5 h, 1 h, 2 h, 3 h), and the water loss rate at each time point was calculated. The experiment was replicated three times. ANOVA was used to evaluate the differences between the wild-type and transgenic plants.

### 4.8. Stomatal Density and Stomatal Aperture

The fourth expanded rosette leaves of Arabidopsis grown in soil for one month were detached and left on the laboratory bench for one hour. Normal and dehydrated leaves were placed in Carnot’s fixative solution (absolute ethanol to glacial acetic acid = 3:1) for 24 h. Then, they were dehydrated with 30%, 50%, 70%, 80%, 85%, 90%, 95%, and 100% alcohol for 30 min each, dehydrated with 100% alcohol again, and placed in a clearing solution (trichloroacetaldehyde to water to glycerol = 8:3:1) until they became transparent. Stomatal apertures were observed under a microscope, and the ratio of stomatal length to width was recorded using ImageJ software. At least 30 stomata of each sample per replicate were measured, and three replicates were performed.

### 4.9. ROS Measurements

Histochemical assays for ROS accumulation were performed using DAB and NBT staining. Arabidopsis leaves were placed in a prepared NBT staining solution (0.01 g of NBT powder was dissolved in 10 mL of 50 mM phosphate buffer), vacuumed for 30 min, and placed in the dark at room temperature for 10 h, and then dipped in decolorizing solution (acetic acid to glycerol to ethanol = 1:1:3) in 95 °C boiling water for 5 min, stored in 95% ethanol, and observed under a stereomicroscope. The detached Arabidopsis leaves were soaked in DAB staining solution (0.1 g/mL DAB, PH 3.8), vacuumed (0.5 MPa) for 30 min, and placed in the dark at room temperature for 10 h. The decolorization method was similar to that of NBT staining. Each line contained at least 12 different seedlings, and representative images are shown.

Quantitative measurement of H_2_O_2_ concentration was performed using the potassium iodide method. Briefly, 100 mg leaf samples were frozen in liquid nitrogen and ground into powder. Then, 1 mL of precooled 0.1% trichloroacetic acid (TCA) solution was immediately added and mixed with the samples. After cryogenic centrifugation (12,000× *g*, 4 °C, 15 min), an equal volume of PBS buffer was added to 500 µL supernatant, then 1 mL of 1 M potassium iodide (KI) solution was added, and the mixture was shaken at 150 rpm for 1 h at 30 °C. The absorbance value was determined at 390 nm wavelength. In addition, the standard curve was made with 300 µmol/L H_2_O_2_. Each experiment was performed in six replicates.

The activities of antioxidant enzymes (SOD, POD, and CAT) were measured following the aforementioned protocols. The units of the antioxidant enzyme activities were defined as follows: a unit of SOD activity is the quantity of enzyme required to cause 50% inhibition of the photochemical reduction in NBT per minute at 560 nm; a unit of POD activity is the amount of enzyme required to cause a 0.01 increase in the absorbance of H_2_O_2_ per minute at 470 nm; and a unit of CAT activity is the amount of enzyme required to cause a 0.01 decrease in the absorbance per minute at 240 nm.

## 5. Conclusions

In conclusion, our study demonstrated that *ZmLBD33* functions as a negative regulator of drought resistance in Arabidopsis, primarily through modulating stomatal aperture and H_2_O_2_ signaling. These findings provide novel insights into the role of Class II *LBD* genes in drought stress responses and highlight their potential as biotechnological targets for improving crop drought tolerance. The identification of *ZmLBD33*′s regulatory mechanism underscores the complexity of drought adaptation strategies in plants. Further studies are warranted to elucidate the molecular mechanisms underlying *ZmLBD33*-mediated regulation of drought-responsive genes and to investigate the functional conservation of this regulatory pathway across different plant species, which could facilitate the development of drought-resilient crops through genetic engineering approaches.

## Figures and Tables

**Table 1:** Cis-elements in the promoter region (~1 kb) of *ZmLBD33*.

Site Name	Sequence	Position	Strand	Function
ABRE	ACGTG	682	+	abscisic acid responsiveness
ABRE	GCCGCGTGGC	761	−	abscisic acid responsiveness
ABRE	ACGTG	624	−	abscisic acid responsiveness
ABRE	CACGTG	681	−	abscisic acid responsiveness
ARE	AAACCA	129	+	anaerobic induction
ARE	AAACCA	806	+	anaerobic induction
ARE	AAACCA	590	−	anaerobic induction
G-Box	CACGTT	624	+	light responsiveness
G-Box	CACGTG	681	−	light responsiveness
G-box	CACGTG	681	−	light responsiveness
Sp1	GGGCGG	492	−	light responsiveness
Sp1	GGGCGG	744	+	light responsiveness
Sp1	GGGCGG	511	−	light responsiveness

“+” corresponds to the coding strand, while “−” denotes the complementary non-coding strand.

## Data Availability

The original contributions presented in this study are included in the article/Supplementary Material. Further inquiries can be directed to the corresponding authors.

## Supplementary Materials

The following supporting information can be downloaded at: https://www.mdpi.com/article/10.3390/plants14091305/s1, Table S1: The primers of RT-qPCR and vector construction.
